# Bilateral Psoas Muscle Abscess Associated with Emphysematous Cystitis

**DOI:** 10.1155/2015/285652

**Published:** 2015-02-10

**Authors:** Jae-Ki Choi, Jae-Cheol Kwon

**Affiliations:** ^1^Department of Internal Medicine, College of Medicine, The Catholic University of Korea, Seoul, Republic of Korea; ^2^Department of Internal Medicine, Chung-Ang University College of Medicine, Seoul, Gyeonggi-do, Republic of Korea; ^3^National Health Insurance Corporation Ilsan Hospital, Gyeonggi-do, Ilsan-si 156-775, Republic of Korea

## Abstract

Psoas muscle abscess associated with emphysematous urinary tract infection is very rare. There were very few reports about urinary tract infections such as renal abscess, perinephric abscess, and emphysematous pyelonephritis complicated with psoas muscle abscess; however, psoas muscle abscess associated with emphysematous cystitis has not yet been reported. Here, we report a case of bilateral posas muscle abscess following emphysematous cystitis in an 81-year-old nondiabetic man, who was treated successfully with prolonged antibiotic therapy and supportive care. Early recognition of psoas muscle abscess can prevent aggressive interventional procedure and warrant good prognosis.

## 1. Introduction

Emphysematous cystitis (EC) and psoas muscle abscess (PsA) are rare disease entities. EC is a complicated lower urinary tract infection (UTI) characterized by air within the bladder wall and lumen. It is most common in middle-aged diabetic women. Predisposing conditions include chronic UTIs, indwelling urethral catheters, urinary tract outlet obstruction, or neurogenic bladders [1].* Escherichia coli* and* Klebsiella pneumoniae* are the predominant etiologic organisms [2]. Clinical spectrum varies from incidental diagnosis on abdominal imaging to severe sepsis. Although most cases could be managed with a combination of antibiotics and bladder drainage, about 10% of EC cases required surgical intervention, including cystectomy or partial cystectomy [3].

The classic clinical triad of PsA is fever, back pain, and limp; however, the clinical presentation of PsA is usually variable and nonspecific [4]. Underlying gastrointestinal tract diseases, especially inflammatory bowel disease, are the most common concomitant diagnosis associated with PsA [5]. Cases of secondary PsA from UTIs were reported from renal abscess, perinephric abscess, and emphysematous pyelonephritis, none from EC [6–8]. The management of PsA generally involves drainage or surgical resection as well as adequate antibiotic therapy. The delayed diagnosis and treatment may cause high morbidity and mortality [9]. Here, we present a rare case of EC followed by bilateral PsA in a nondiabetic old man who was treated successfully with medical management.

## 2. Case Report

**Figure 1:**
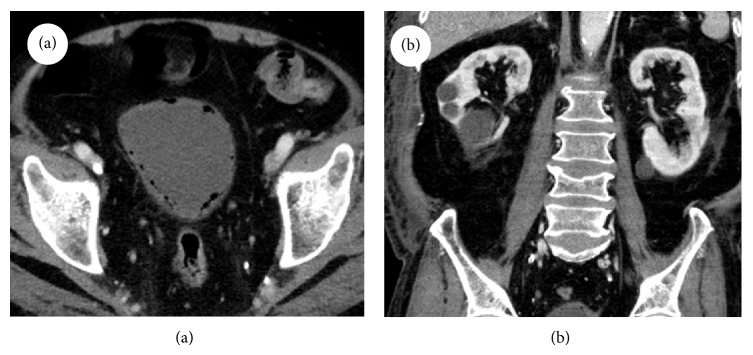
(a) Computed tomography of the abdomen showed multiple diffuse gas collection in the bladder wall suggestive of emphysematous cystitis. (b) Both kidneys and psoas muscle were unremarkable except multiple cortical cysts.

An eighty-one-year-old man presented to the emergency department due to fever and shortness of breath. He had sustained a nonpenetrating injury to his left flank by colliding with the bed corner approximately 2 weeks earlier. His past medical history was notable for 12 years of hypertension and he underwent right hemicolectomy due to colon cancer 7 years ago. He was a nonsmoker and did not drink alcohol. On physical examination, the patient was alert and acutely ill-appearing. His vital signs were blood pressure, 90/60 mmHg; pulse rate, 132/min; respiratory rate, 23/min; and body temperature, 38.1°C. The abdomen was distended and bowel sound was decreased. There was mild tenderness over his suprapubic and lumbar area. Laboratory tests revealed the following: white blood cell (WBC) count, 10,880/mm^3^ with 85.7% neutrophils; hematocrit, 48.2%; hemoglobin, 17.7 g/dL; platelet count, 42,000/mm^3^; blood urea nitrogen, 39.1 mg/dL; serum creatinine, 1.05 mg/dL; sodium, 146 mmol/L; potassium, 3.3 mmol/L; chloride, 116 mmol/L; total protein, 4.5 g/dL; albumin, 2.0 g/dL; aspartate transaminase, 134 IU/L; alanine transaminase, 52 IU/L; erythrocyte sedimentation rate, 11 mm/hr; C-reactive protein (CRP), 20.0 mg/dL; prothrombin time, 12.3 seconds (INR 1.19); activated partial thromboplastin time, 33.2 seconds; fibrinogen, 533 mg/dL; antithrombin III 58%; FDP, >20 ug/mL; and lactic acid 3.15 mmol/L. The urinalysis revealed protein 2+, glucose 1+, nitrite positive, red blood cells 3+/HPF, and WBC 3+/HPF. The arterial blood gas analysis showed pH 7.54, PaCO_2_ 24 mmHg, PaO_2_ 77.5 mmHg, and oxygen saturation 96.6%. Plain chest and abdominal radiography was unremarkable. Computed tomography (CT) scan of the abdomen and pelvis revealed multiple diffuse gas collection along the bladder wall and multiple cortical cysts in both kidneys (Figure 1). Other organs were unremarkable. These results were suggestive of severe sepsis caused by emphysematous cystitis.

**Figure 2:**
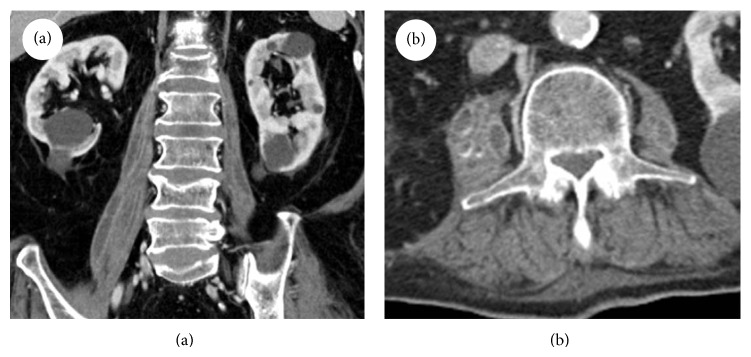
Follow-up abdominal computed tomography showed bilateral multiple abscess formation in the psoas muscle. (a) Frontal plane, (b) Transverse plane.

Vancomycin (1.0 g q12 h) and meropenem (1.0 g q8 h) were administered for empirical antibiotic therapy. Bladder catheterization was delayed due to urethral stricture. It could be done only after incision of urethral opening by the urologist. Intravenous immunoglobulin was administered concurrently as an adjuvant therapy for severe sepsis. Urine culture was negative but blood culture grew extended-spectrum *β*-lactamase nonproducing* K. pneumoniae* which was identified by automated Vitek2 system. This organism was sensitive to amikacin, aztreonam, cefotaxime, ceftazidime, ciprofloxacin, piperacillin/sulbactam, and imipnemem but resistant to ampicillin. Meropenem was changed to levofloxacin (500 mg q24 h). The patient's condition deteriorated with high fever, tachypnea, hypoxemia, and shock. Endotracheal intubation was done on hospital day (HD) 10. Follow-up CT of the abdomen on HD 12 showed bilateral multiple abscess formation in the psoas muscle (Figure 2) which did not exist on the initial imaging study. Vancomycin and levofloxacin were changed to teicoplanin (400 mg q12 h three times and then 400 mg q24 h) and meropenem; however, fever was sustained and CRP was kept high. We stopped meropenem and teicoplanin and started tigecycline (100 mg one time and then 50 mg q12 h) on HD 23. The patient's condition was improved and endotracheal tube was removed on HD 26.

**Figure 3:**
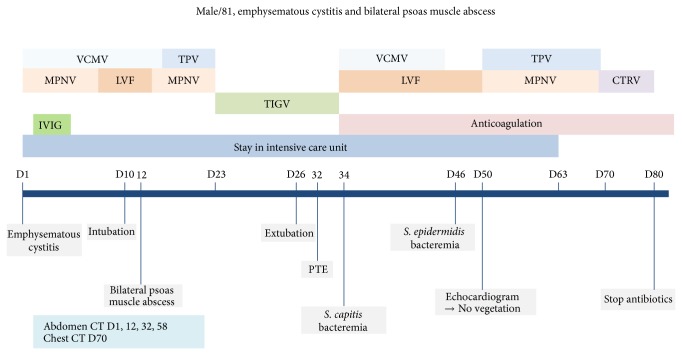
Timeline for clinical events, radiology, and antibiotic treatment. VCMV, vancomycin; TPV, teicoplanin; MPNV, meropenem; LVF, levofloxacin; CTRV, ceftriaxone; TIGV, tigecycline; IVGV, intravenous immunoglobulin; PTE, pulmonary thromboembolism; CT, computed tomography.

Afterwards pulmonary thromboembolism and bacteremia caused by methicillin resistant cogaulase negative staphylococci developed during hospitalization which were treated successfully with anticoagulation and glycopeptides. Follow-up CT scan on HD 58 showed resolution of bilateral psoas muscle abscess and emphysematous cystitis. The patient was moved to general ward from medical intensive care unit (ICU) on HD 63. Antibiotics were stopped on HD 80 and he was discharged on HD 99. The clinical course and events were described in Figure 3.

## 3. Discussion

Complicated UTIs such as EC are predisposed to patients with chronic UTIs, indwelling urethral catheters, neurogenic bladders, or urinary tract outlet obstructions [1]. The diagnosis can be made radiologically by simple plain film or CT scan, through direct visualization on cystoscopy, or pathologically on tissue from bladder biopsy or autopsy. Etiologic organisms are usually identified by urine or blood cultures. The present case was diagnosed with CT scan.* K. pneumoniae* grew from blood but not from urine. Delayed bladder catheterization due to urethral stricture made it impossible to conduct urine culture before antibiotic use. This is why the urine culture was negative. Meropenem had been injected 2 times before successful urethral catheterization.

Recent increase in reported cases is mainly due to a wider use of abdominal imaging and a greater awareness of this unusual disease. Thomas et al. analyzed 135 EC cases published in English between 1956 and 2006 [3]. Sixty-six cases (49%) were reported within the recent 15 years. The median age was 66 years, women consisted of 64%, and 37% had diabetes mellitus (DM).* E. coli* (58%) was the most prevailing causative organism followed by* K. pneumoniae* (21%),* Enterobacter aerogenes *(7%), and* Clostridium perfringens* (6%). Most patients (90%) were treated with medical treatment, including a combination of antibiotics, bladder drainage, and glycemic control. The overall mortality rate in this review was 7%. Although 14 cases (10%) were reported to have another gas-forming urinary tract infection, none of them had PsA. Our patient was a nondiabetic man of old age but had urinary stasis secondary to bladder outlet obstruction which might be predisposed to EC. Bilateral PsA was developed as a complication.

PsA is usually classified as primary or secondary on the basis of the presence or absence of underlying disease. Primary PsA is probably caused by hematogenous spread of an infectious process from an occult source in the body. The underlying conditions include DM, intravenous drug abuse, renal failure, and immunosuppression [4]. Meanwhile, secondary PsA is usually caused by contiguous spread of infectious process from adjacent organs. The gastrointestinal tract diseases, mainly Crohn's disease, are the main cause of secondary PsA. Others include vertebral osteomyelitis, urinary tract infections, and intra-abdominal malignant tumors [5, 10]. Renal abscess, perinephric abscess, and emphysematous pyelonephritis are reported predisposing UTIs associated with PsA [3, 6–8]. Also, bilateral PsA was reported to be less than 3% of all PsA [11, 12]. This is the first case of PsA associated with EC. We suppose that this PsA resulted from hematogenous spread of* K. pneumonia* because of intact perivesicular lining, normal ureter, and preserved kidney capsules as well as bilaterality of PsA. The abundant blood supply of the psoas muscle is likely to permit hematogenous spread [10]. The absence of no psoas pathology at admission and presence of bilateral PsA on HD 11 further support our assumption.

The clinical presentation of PsA is often variable and nonspecific. The classical clinical triad of fever, back pain, and limp is present in only 30% [4]. As the psoas muscle is innervated by L2–4, pain can radiate to hip and thigh. Other symptoms are vague abdominal pain, malaise, nausea, and weight loss. Our patient had fever and mild lumbar tenderness. Despite broad spectrum antibiotic therapy and aggressive supportive care in ICU, the patient's condition deteriorated. We conducted abdomen-pelvis CT again to check the status of preexisting EC or other intra-abdominal comorbidities; then, we found bilateral PsA which did not exist in the initial evaluation.

The management of PsA generally involves the use of appropriate antibiotics along with percutaneous or surgical drainage [4, 10]. Some researchers emphasized PsA size in the choice of initial therapy [13]. They suggested that PsA that are smaller than 3 cm in greatest diameter may be managed successfully with antibiotics alone [13]. Eighteen primary and 23 secondary PsA cases were included in that study. Treatment was via open drainage (*n* = 1, 3%), CT-guided percutaneous drainage (*n* = 26, 63%), or antibiotics alone (*n* = 14, 34%). Statistical analysis showed that the median size of PsA in the percutaneous group was significantly larger than in the antibiotics group (6 versus 2 cm; *P* < 0.001). Our case had bilateral diffuse lesions and was treated successfully with medical management alone.

The reported mortality rates are 2.4% in primary PsA and 19% in secondary PsA [4]. The mortality rate in untreated patients is almost 100% [11]. Early diagnosis is the key for minimizing invasive procedure and favorable outcome.

In summary, the present case shows the importance of early diagnosis and treatment of PsA. The clinician should keep in mind that PsA can develop as a complication of bacteremia which is not uncommon in community acquired urinary tract infections.
